# Two cases of agenesis of the dorsal pancreas and a review of the literature

**DOI:** 10.1186/s12876-020-01245-8

**Published:** 2020-04-06

**Authors:** Wentong Mei, Feng Cao, Fei Li

**Affiliations:** 1 Department of General Surgery, Xuanwu Hospital, Capital Medical University, No.45 Changchun Street, Beijing, 100053 China; 2 Clinical Center for Acute Pancreatitis, Capital Medical University, Beijing, China

**Keywords:** Agenesis of the dorsal pancreas (ADP), Pancreatic disease, Pancreatic dysplasia, Case report

## Abstract

**Background:**

Agenesis of the dorsal pancreas (ADP) is a very rare disease with no specific symptoms, and the pathogenesis is not clear. Some patients will be accompanied by other diseases, such as pancreatic tumor or pancreatitis. But most cases are very atypical and difficult to distinguish. Some syndromes of pancreatic exocrine insufficiency are common in patients with ADP. Here, we report two cases of ADP and summarize the clinical features, diagnosis, and treatment of ADP.

**Case presentation:**

Case A is a 65-year-old Chinese woman who presented with abdominal pain accompanied by nausea, bloating and acid reflux. The enhanced abdominal CT scan found nothing meaningful except the absence of the body and tail of the pancreas. The diagnosis was considered as gastrointestinal dysfunction cause by exocrine pancreatic insufficiency and recovered after symptomatic treatment. Case B is a 61-year-old Chinese woman who presented with abdominal pain accompanied by fever, vomiting and bloating. The abdominal CT showed multiple stones in the gallbladder, and the body and tail of the patient’s pancreas were absent. She was diagnosed with cholelithiasis and recovered after laparoscopic cholecystectomy.

**Conclusion:**

Agenesis of the dorsal pancreas (ADP) is a rare congenital disease with an unclear pathogenesis that presents multiple symptoms. It should be considered when the patients have non-specific, persistent and unexplained symptoms such as bloating or uncontrolled blood sugar. Imaging examination is helpful for diagnosis. And it does not require surgical intervention unless it accompanies other diseases, EPI need to be considered when the non-specific gastrointestinal symptoms appear.

## Background

Agenesis of the dorsal pancreas (ADP) is a very rare anatomical variation of the pancreas. It is characterized by the partial or total loss of the body and tail of the pancreas. The disease has no specific clinical symptoms but may accompany pancreatic secretion insufficiency and other pancreatic diseases. Most cases were discovered by chance. This paper reports two cases of ADP, summarizes the clinical features, diagnosis and treatment of the disease, and reviews the latest literature.

## Case presentation

### Case A

A 65-year-old Chinese woman presented with a history of abdominal pain for 3 days. The patient developed paroxysmal abdominal pain in the past 3 days, accompanied by nausea, bloating and acid regurgitation. The pain was limited to the middle and upper abdomen, and anal exhaust and bowel movements were normal. The symptom did not become aggravated during the 3 days. She denied the symptoms of steatorrhea. The patient has had type 2 diabetes for more than 20 years and used a drug regimen to control the DM, but the patient complained that her fasting blood glucose was still poorly controlled. She had no other diseases. Her family medical history was unremarkable.

**Fig. 1:**
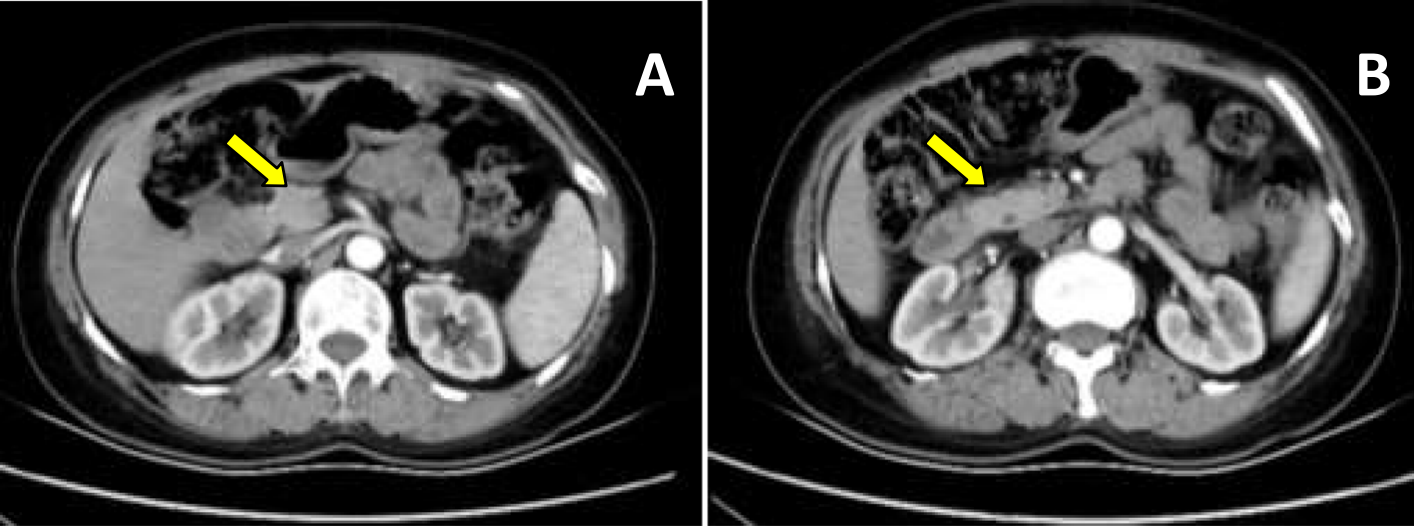
**a** and **b** Axial arterial phase CT image showing the absence of the pancreatic body and tail, without pancreatitis and pancreatic duct dilatation. The *arrow* show the pancreatic head

No positive signs were found in the physical examination. The results of the hematological examinations were normal. Only the blood sugar (11.7 mmol/L) and triglycerides (4.3 mmol/L) were abnormal in the blood biochemical examination, and no signs of infection were found. No abnormal results were observed from the electronic gastroscopy and electronic colonoscopy, which were performed 2 months ago. An enhanced abdominal CT scan revealed slightly increased gastrointestinal contents and no signs of acute abdominal (obstruction, perforation, etc.) or celiac vascular disease but found that the patient did not have the body and tail of the pancreas (Fig. 1a-b).

So the preoperative diagnosis is gastrointestinal dysfunction, agenesis of the dorsal pancreas, and pancreatic exocrine insufficiency were not excluded. Mosapride and trimebutine were used to improve gastrointestinal function, and 0.6 g pancreatin enteric-coated capsules were given to the patient before each meal continuously to supplement her pancreatic exocrine function. And Intermediate effect insulin were used to help control the fasting blood glucose.

The patient returned to the gastrointestinal surgery clinic 1 week later, complaining that although the symptoms were significantly relieved, but there was still mild postprandial bloating. Here fasting blood glucose fluctuates from approximately 5-8 mmol/L. The physician continued to give the patient the above medications.

### Case B

A 61-year-old Chinese woman presented with a history of upper right abdominal cramping with vomiting and fever for 10 h. The patient developed the above symptoms after eating greasy food 10 h ago; she vomited her stomach contents and bile repeatedly, but did not experience hematemesis. Fever occurred after the abdominal pain, and the highest body temperature was 38.8 °C. She did not present with chills, jaundice, constipation or diarrhea. Apart from these observations, the patient had a history of intermittent bloating after meals for at least 20 years, and she denied the symptoms of steatorrhea. The patient has had type 2 diabetes for 12 years, and metformin and glimepiride were used to control the DM; her fasting blood sugar level fluctuates from approximately 7–10 mmol/L. She also has congenital scoliosis. Her family medical history was unremarkable.

**Fig. 2:**
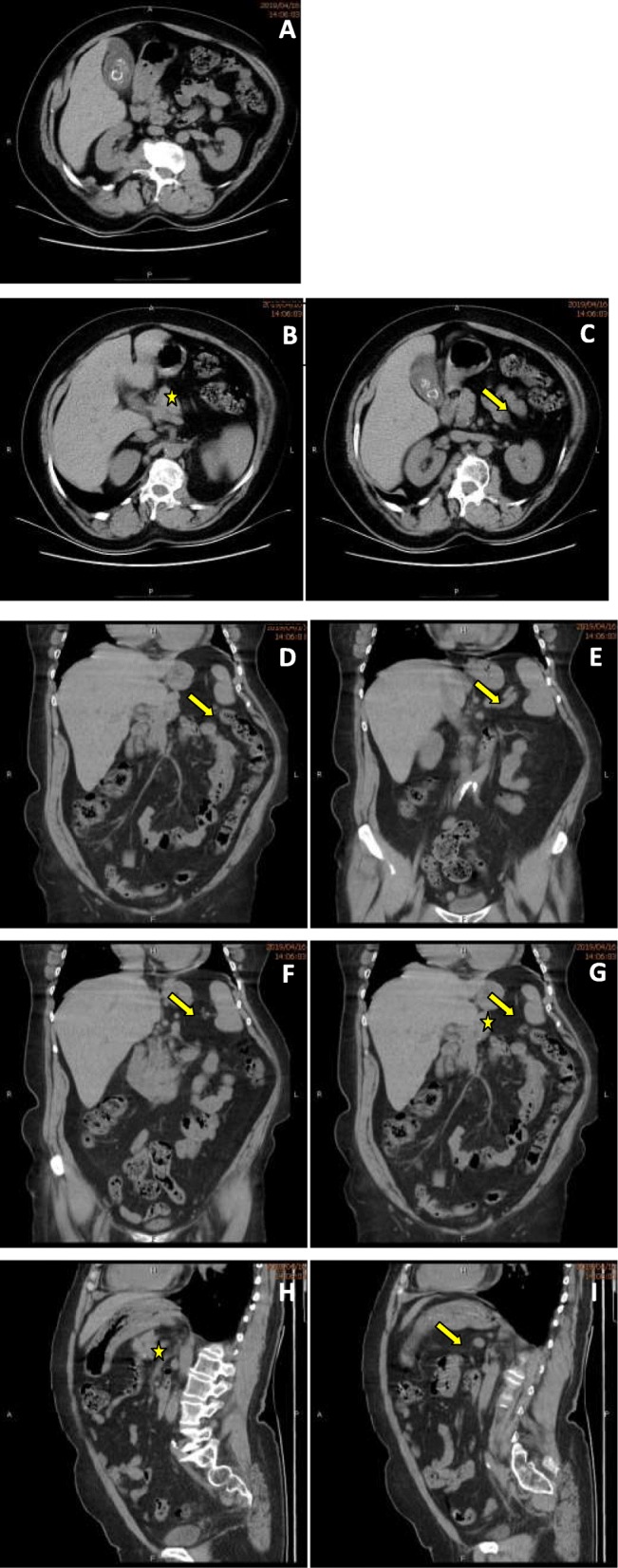
**a** Abdominal CT image shows the stones in the gallbladder and the inflamed gallbladder. **b**-**i** Abdominal CT image including coronal reconstruction (**d**-**f**) and sagittal reconstruction (**h** and **i**) image shows the head of the pancreas without the body and tail. The *star* shows the pancreas head, the *arrow* indicates the area of the body and tail of the pancreas and now only the retinal tissue and the blood vessels of the spleen is there

The patient’s body temperature was 38.9 °C, and her BMI was 32 kg/m^2^. The physical examination revealed mild abdominal muscle tension, tenderness in the right upper quadrant, and positive Murphy’s sign. The hematological examinations showed a high white blood cell count (14.4*10^9 /L) and an elevated neutrophil percentage (87.7%). The blood biochemistry tests showed high blood sugar (12.1 mmol/L), low K^+^ (2.9 mmol/L) and low total cholesterol levels (6.2 mmol/L). The abdominal CT including coronal reconstruction and sagittal reconstruction image showed multiple stones in the gallbladder, and the gallbladder wall was rough and had mild edema (Fig. 2a). The body and tail of the patient’s pancreas were absent; in the area where the tail of the pancreas should be, we only observed blood vessels of the spleen (Fig. 2b-i). Since the patient had no evidence of bile duct stones, MRCP was not performed in the emergency department.

So the preoperative diagnosis is acute cholecystitis, cholelithiasis, agenesis of the dorsal pancreas, and pancreatic exocrine insufficiency were not excluded. We gave the patient gastric decompression. Cefminox were used to control the infection, and magnesium sulfate was used as a cholagogue. In addition, to relieve her symptoms, we used anisodamine, metoclopramide and famotidine. Rehydration therapy and potassium supplementation were conducted simultaneously. After the patient recovered from the acute stage, she underwent laparoscopic cholecystectomy; 1.8 g pancreatin enteric-coated capsules were given per day continuously to relieve the symptoms of pancreatic exocrine function.

The patient’s symptoms completely improved after the drug regimen in the acute stage, and she had no abdominal pain after the LC surgery. After 3 weeks of pancreatic enzyme replacement therapy, she said that her symptoms of bloating after meals significantly improved.

## Discussion and conclusion

The pancreas is formed by the ventral and dorsal pancreatic buds. Most of the pancreas is derived from the dorsal pancreas, which differentiates into the body and tail of the pancreas, part of pancreatic head, accessory pancreatic duct and distal part of the pancreatic duct [1].

Agenesis of the dorsal pancreas (ADP) is a rare anatomical variation of the pancreas. It was first reported in 1911 by Schnedl, and approximately 100 cases have been published up to now [2]. ADP is characterized by the absence of the pancreatic body and tail; if only the pancreatic tail is missing, it is called partial agenesis of the dorsal pancreas. If the pancreatic body is also lacking and only the pancreatic head is present, it is known as complete agenesis of the dorsal pancreas [3]. At present, the pathogenesis of this disease is not fully understood. The HNF1B gene is known to regulate pancreatic development, some studies have found that ADP and pancreatic exocrine dysfunction are parts of the phenotype in HNF1B mutation carriers, and GATA6 gene mutations also can lead to pancreatic hypoplasia [4–6]; animal experiments have confirmed that retinaldehyde dehydrogenase 2 (Raldh2) [7] and gene H1xb9 [8] mutations can cause ADP in mice.

Most simple ADP patients have no specific symptoms. Patients can be found through imaging examinations for common abdominal symptoms such as pain or bloating. There is no evidence that ADP alone can cause abdominal pain. There were several studies that reported that ADP can be complicated by acute pancreatitis. The mechanism of which may be Oddi sphincter dysfunction, pancreatic head compensatory hypertrophy, increased pancreatic juice secretion, and pancreatic duct hypertension [9, 10]. Because of the above mechanism, the possibility of ADP combined with chronic pancreatitis has theoretically increased. A small number of patients have been diagnosed with chronic pancreatitis due to persistent chronic abdominal pain, and ADP has been subsequently discovered [11]. Moreover, we have searched a limited number of ADP-related cases of recurrent pancreatitis. One case reported recurrent abdominal pain after drinking [12]. Amylase and lipase values did not support the diagnosis of acute pancreatitis and the symptoms was disappeared after forbearing from alcohol; Another case was also recurrent abdominal pain [13]. Endoscopy revealed that Santorini’s duct was dilated and contained calculi but Wirsung’s duct was nearly normal. And the junction between the two ducts was slightly narrowed. It was considered chronic pancreatitis. The patient underwent internal endoscopic sphincterotomy and the symptoms were relieved after the operation. In addition to most of the islet β cells being located in the tail of the pancreas, patients with ADP also have diabetes due to insufficient insulin secretion, but not necessarily abdominal symptoms [14, 15]. Approximately half of these patients need insulin therapy. A young woman with ADP was reported to have asymptomatic insulin-dependent diabetes mellitus and already had severe retinal lesions at the time of presentation [15]. Other studies reported ADP combined with ampullary tumors, and the pathological types included cystic adenocarcinoma, solid pseudopapillary tumors, intraductal papillary mucinous neoplasms, neuroendocrine tumors and cholangiocarcinoma [16, 17]. Some patients with pancreatic pseudocysts were also found [18].The surgical treatment of these types of patients is no different from that for patients with normal pancreatic development, but after pancreatic head resection, due to the lack of the tail of the pancreas, postoperative pancreatic exocrine insufficiency and exogenous insulin dependence will be needed. The association between tumorigenesis and ADP is unclear, but chronic pancreatitis caused by the latter is indeed one of the risk factors for cancer. In addition, ADP associated with other organ malformations has also been reported, including polycystic kidney disease, Kartagener syndrome, multiple splenic deformities, congenital choledochal cysts and biliary atresia [19, 20].

The diagnosis of ADP mainly relies on imaging examinations. Schnedl supported that the loss of the accessory pancreatic duct and the absence of the tail of the pancreas are conditions for the diagnosis of complete dorsal pancreatic hypoplasia. As most ADP patients are accidentally discovered because of abdominal pain, they mainly first undergo ultrasound and CT. Ultrasound is affected by the gas in the intestine so it is difficult for this modality to identify the part of pancreas near the splenic vessels; thus, it is easy for ultrasound to miss the disease. CT and MRI can show the complete or partial disappearance of the pancreatic body and tail [21]. The splenic vessels are only visible in that area, and the pancreatic head could be compensated and slightly full. ERCP/MRCP can clearly show the shape of the main and accessory pancreatic ducts and has high value for identifying the complete or incomplete types of ADP [21, 22]. The disease needs to be differentiated from pancreatic fat infiltration, chronic pancreatitis and pancreatic body and tail atrophy. According to previous literature and our experience, ADP mostly manifests as the absence of the pancreatic body and tail, the density and morphology of the remaining pancreatic head should be normal, except with compensatory enlargement of pancreatic head. Pancreatic fat infiltration have unique echo characteristics in ultrasound examination and fat signal fraction (FSF) is increased in MR image, it shows more severe signal suppression than pancreatic tissue on fat suppression imaging; While chronic pancreatitis mostly has pancreatic duct dilatation, pancreas calcification and a lower apparent diffusion coefficient (ADC) values in MR image, which means an increase in fibrosis. The full pancreatic head needs to be differentiated from pancreatic head tumor and pancreatitis. By observing the peripancreatic fat gap, the presence of calcifications and enlarged lymph nodes can help to confirm the diagnosis.

There is no special treatment for ADP itself, but sometimes it is necessary to treat the accompanying disease. In case A, we did not see clear positive signs during the physical examination, and no new observations were found in the auxiliary examination, but the patient had obvious gastrointestinal symptoms. Therefore, we consider that this is a functional disease. The symptoms of patients are significantly improved after taking pancreatin enteric-coated capsules, which suggests that ADP may cause patients to have symptoms related to exocrine pancreatic insufficiency(EPI). Such cases are very rare in previous literature reports [23, 24]. Combining our case and previous reports, pancreatin enteric-coated capsules is helpful to relieve symptoms. The patient in Case B also has persistent symptoms of EPI, but we are more concerned about her suffering from acute cholecystitis with gallstones and ADP. According to previous studies, such case are also rare. We try to demonstrate that even in the presence of ADP, many symptoms may not be related to the disease via these cases’ study, and no special treatment of ADP is needed in such circumstances. In our cases, the patient has diabetes, which may be related to the loss of islet cells on the dorsal pancreas, so exogenous insulin can be considered in cases where the drug controls poor blood glucose. For those who need surgery, we believe that in addition to performing the aforementioned differential diagnosis to clarify the surgical indications, it is necessary to identify the presence of other anatomical variations, including ADP, in the surgical field for the intraoperative strategy. However, if pancreatectomy is required, the volume of the pancreas remaining after surgery should be considered.

These two patients ADP had nonspecific symptoms and were accidentally discovered, and both had bloating and diabetes, which may be related to ADP. Some tests to diagnose pancreatic exocrine insufficiency may be useful. It is regrettable that our hospital has not carried out relevant tests, so this part of the data is not complete. However, since the patients’ illnesses were not related to the pancreas and bile duct, no MRCP test was performed, and the state of the pancreatic duct could not be identified. Therefore, we believe that if ADP patients are sporadically found and do not have pancreas-related symptoms, from the perspective of health economics, whether further examinations are needed to determine the type of ADP remains to be discussed. After all, there is no special treatment for this disease.

In summary, ADP is a rare type of autosomal genetic disease. Non-specific, persistent and unexplained symptoms such as bloating or uncontrolled blood sugar may be associated with this disease. Imaging examination is helpful for diagnosis. Simple ADP does not require special treatment; if it accompanies other diseases and doctors can make a clear diagnosis with imaging examinations, the patients can benefit from a differential diagnosis, drug treatment (such as insulin use) and surgery.

## Data Availability

All data generated or analyzed during this study are included in this published article.

## Abbreviations

ADP: Agenesis of the dorsal pancreas
MRCP: Magnetic Resonance Cholangiopancreatography
